# Exploring Spike-Dependent and ACE2-Independent SARS-CoV-2 Interactions with Salivary Epithelial Cells in the Absence of ACE2

**DOI:** 10.3390/biology15100778

**Published:** 2026-05-14

**Authors:** Caitlynn M. L. Barrows, Thaise C. Geremias, Simon Young, Mary C. Farach-Carson

**Affiliations:** 1 Katz Department of Oral and Maxillofacial Surgery, UTHealth Houston School of Dentistry, Houston, TX 77054, USA; cmbarrows@houstonmethodist.org (C.M.L.B.); simon.young@uth.tmc.edu (S.Y.); 2 Department of Diagnostic and Biomedical Sciences, UTHealth Houston School of Dentistry, Houston, TX 77054, USA; thaise.c.geremias@uth.tmc.edu

**Keywords:** salivary gland, ACE2, SARS-CoV-2, spike protein, COVID-19

## Abstract

Salivary gland infection by SARS-CoV-2 requires viral entry via routes and mechanisms that remain unresolved. This study examined the expression of the angiotensin-converting enzyme 2 (ACE2) receptor in salivary tissues and basal cell-derived human salivary progenitor cells (hS/PCs), an unstudied potential entry point for SARS-CoV-2. Multiple detection modalities, including immunocytochemistry, Western blotting, flow cytometry and RT-PCR, demonstrated a consistent lack of ACE2 protein and transcript in both tissue specimens and primary salivary epithelial cells. Antigen retrieval at pH 9 was determined to be optimal for immunodetection protocols, yet ACE2 remained undetectable. Small intestine tissue served as a positive control, confirming the validity of the methods and reagents we used. Considering there can be other receptors for SARS-CoV-2, flow cytometric analyses demonstrated that recombinant SARS-CoV-2 spike protein failed to bind to salivary epithelial cells, in contrast to HEK293 cells engineered to overexpress ACE2, which showed robust spike binding. Additional studies showed that patient-derived salivary cells, negative for ACE2, are not infected by the SARS-CoV-2 pseudovirus, while ACE2-positive cells are readily infected. These findings strongly support our conclusion that salivary cells do not serve as major targets for SARS-CoV-2 infection via ACE2, spike protein, or an alternate receptor. Thus, salivary cells are unlikely major targets for SARS-CoV-2 infection, either through direct exposure to viral particles in ductal fluids or via access to basal cells across the basement membrane.

## Simple Summary

The virus responsible for the COVID-19 pandemic, Severe Acute Respiratory Syndrome Coronavirus 2 or SARS-CoV-2, can infect the upper respiratory system through viral spike protein binding to a cell receptor called angiotensin-converting enzyme 2 (ACE2) that is present on the surfaces of target cells. SARS-CoV-2 infects the nasal passages, lungs, and digestive system by binding to ACE2. It has been speculated that the virus can enter the salivary glands as an alternative site of infection where it could spread through saliva droplets. The exact way by which SARS-CoV-2 infection of salivary glands could occur is unknown. Some early studies in the pandemic suggested salivary infection occurred via ACE2 in salivary glands. Other studies implicated alternate receptors. During our thorough investigation using patient-derived salivary gland tissues and cells from patient donors, we found no evidence for ACE2 presence in salivary glands. Further, we could not show direct binding of spike protein to salivary cells, nor detect infection of ACE2-negative salivary cells by the SARS-CoV-2 pseudovirus. We conclude that salivary cells are not likely to be major targets for initial infection by SARS-CoV-2 either by ACE2 or an alternate receptor route.

## 1. Introduction

In early 2019, Severe Acute Respiratory Syndrome Coronavirus 2 (SARS-CoV-2) broke out in Wuhan, China, leading to the global pandemic known as COVID-19 [1]. During its spread from Wuhan across the globe, SARS-CoV-2 acquired significant mutations regularly associated with increased virulence and evasion of the immune system [2]. The most significant of these mutations occurred in the viral spike protein, the site of contact and entry of the virus into human cells [3]. The transmission mode was identified as person-to-person through contact with aerosols and droplets containing the respiratory virus [4]. This transmission method indicates that the upper respiratory system specifically has high involvement in viral spread. Given the similarities between SARS-CoV and SARS-CoV-2, the angiotensin-converting enzyme 2 (ACE2) receptor was quickly identified as the primary site for SARS-CoV-2 binding and became the accepted receptor for viral entry [5,6]. Consequently, several studies explored the tissue locations of ACE2 to identify the potential impact of SARS-CoV-2 on various organs and additional possible sites of direct infectivity beyond the lung [7,8]. Studies implicated the salivary glands as a potential site of infectivity based on bulk RNA sequencing data obtained from publicly available databases including GTEx and Fantom 5, which indicated that the salivary glands had similar transcript levels of ACE2 as compared to the lung [9,10]. At the time of publication, the nasopharyngeal swab was the gold standard for SARS-CoV-2 detection and remains the preferred way of testing, although saliva-based diagnostics have been developed [11,12].

The presence of active viral particles in saliva suggested a role for salivary glands in disease transmission through expelled salivary droplets, in addition to those originating in the nasopharynx or respiratory system [13]. If direct infection of salivary glands occurred, then the pathway by which the virus reaches the salivary epithelium, where any receptors associated with infection would be located, must be deduced. Given their locations and abundance throughout the oral cavity, the 800–1000 minor salivary glands throughout the oral cavity were given initial consideration as sites of primary infection, and some reports demonstrated presence of viral particles present in these minor glands [14]. Despite this observation, it is not entirely clear how the SARS-CoV-2 virus, if present in the oral cavity, would reach the surface and infect these minor glands in titers high enough for general infection. Minor glands present beneath the oral mucosa (10% of total salivary production) lack long, named ducts like those in the major glands, and only possess very short excretory ducts 10–50 µ in diameter that produce a mucous-rich secretion that protects the glands from general infection [15,16]. These microscopic ducts open directly onto the mucosal surface, often within or just beneath the epithelium where they lubricate the oral cavity. The SARS-CoV-2 virus, 100–140 nm in diameter covered with 20 nm spike proteins [17], is small enough to penetrate the excretory ducts, but would need to actively move through mucous secretions in a direction retrograde to fluid flow from the minor glands.

The structure of the major exocrine salivary glands includes terminal end buds consisting of serous and mucosal acini, present in various proportions in different glands, which produce the saliva. Saliva produced by acinar cells is transported and buffered by an organized network of ductal cells before being secreted into the oral cavity. As the larger duct patents in the oral cavity approach the terminal endbuds where saliva is produced, they narrow and branch. Larger interlobular ducts give way to small intralobular/striated ducts and finally to intercalated ducts that conduct saliva from secretory acini to striated ducts. The intercalated ducts of the major salivary glands are the smallest ducts in the ductal system and serve as the initial conduit for saliva produced by acinar cells. Intercalated ducts play important roles in both ion transport and antimicrobial defense [18,19]. Their cellular architecture, including the presence of basal cells, consists of a simple, contiguous cuboidal epithelium with regularly spaced basal cells that rest beneath the cuboidal cells and abut the basement membrane separating epithelium from the underlying stromal compartment. The basal cell population is key for ductal regeneration and repair and expresses markers including K5, K14, and p63 [20]. Our laboratories have studied this cell population for years, having extracted them from both major and minor glands [21,22] and shown them to be a rich stem, progenitor population for tissue engineering and regeneration that we term human stem/progenitor cells (hS/PCs) [20,23]. Relative to studies of SARS-CoV-2 infection of salivary glands, these cells are of high interest as their infection could have the ability to be propagated throughout the gland, unlike that of the terminally differentiated cuboidal ductal cells lining the ducts. Reports have indicated that basal cells present in the ducts express both ACE2 and TMPRSS2, the co-receptor for SARS-CoV-2 [24]. Because of their proximity to stroma and the vascular system of the salivary gland, salivary basal cells could conceivably be infected by viral particles in circulation spreading from other sites of major SARS-CoV-2 infection. Entry through this route would be unlikely to involve ACE2, but could involve an alternate receptor, integrins α5β3 and α5β1 [25,26], demonstrated to facilitate entry of SARS-CoV-2 [27], presumably from circulation through a compromised basement membrane [28]. These integrins have previously been shown to be expressed in salivary epithelial cells [29,30,31].

In this work, we set out to determine systematically where ACE2 is localized in the complex structures of the salivary gland, which cells could be targeted by SARS-CoV-2 via an ACE2-dependent pathway and whether basal cells could be recognized by spike protein. This information could shed new light on the routes to infectivity of salivary cells by coronaviruses.

## 2. Materials and Methods

### 2.1. Cell Culture

Primary cells were isolated from salivary glands. All procedures for tissue collection followed approved Institutional Research Board guidelines at UTHealth Houston. Patients provided informed consent, and all sample collections were deidentified except for sex and age. Human parotid and human minor salivary glands were processed as previously described [21,22]. Briefly, salivary glands were washed in 1X PBS without calcium and magnesium. Glands were then washed in cold DMEM/F12 (Gibco, Waltham, MA, USA) supplemented with 1% *v*/*v* penicillin–streptomycin, 1% *v*/*v* amphotericin B, and 1% *v*/*v* betadine solution (from 10% povidone iodine stock). A final wash used cold DMEM/F12 supplemented with 1% *v*/*v* penicillin–streptomycin and 1% *v*/*v* amphotericin B. All solutions were sterile-filtered through a 0.22 µm PES filter. Glands were minced with scissors to create a slurry and plated in a T-75 flask with 5 mL of William’s Medium E without L-glutamine (Sigma, St. Louis, MO, USA, W4128) supplemented with 1 mg/mL albumin from human serum (Sigma, A1887), 1% *v*/*v* CTS™ GlutaMAX™-I Supplement (Gibco, A12860-01), 1% *v*/*v* penicillin–streptomycin, 1% *v*/*v* insulin–transferrin–selenium (InVitria, Junction City, KS, USA, 777ITS032), 0.1 µM dexamethasone, and 10 ng/mL human epidermal growth factor (Gibco, PHG0311). Cultures were left for 5 days to allow for full adherence of explants to the cell culture flask before media was changed. After 5–7 days, the first passages were conducted with 0.125% (*v*/*v* from stock) trypsin-EDTA.

Additional cell lines, HEK293T (ATCC, Manassas, VA, USA, CRL-3216) and ACE2-HEK293T (Genecopoeia, Rockville, MD, USA, SL221), were cultured in DMEM high glucose (Corning, Corning, NY, USA, 10-013-CV) supplemented with 10% *v*/*v* FBS and 1% *v*/*v* penicillin–streptomycin. ACE2-HEK293T medium was supplemented with 100 µg/mL hygromycin-B. CACO2 (ATCC, HTB-37) cells were cultured in EMEM (ATCC, 30-2003) supplemented with 20% *v*/*v* FBS and 1% *v*/*v* penicillin–streptomycin. All cultured cells were maintained in an incubator at 37 °C with 5% CO_2_.

### 2.2. Immunocytochemistry

For immunostaining of cells grown in 2D monolayer cultures, samples were washed with 1x PBS (without calcium and magnesium), then fixed with 4% (*v*/*v*) paraformaldehyde (PFA) for 10 min at room temperature (RT) and washed 3X with 1X PBS. Subsequently, samples were permeabilized in 0.3% (*v*/*v*) Triton X-100 (Thermo Scientific, Waltham, MA, USA, A16046AP) for 4 × 5 min with agitation and blocked with 10% (*v*/*v*) goat serum pH 7.4 diluted in 0.3% (*v*/*v*) Triton X-100 for 1 h at RT with agitation. Samples were incubated with anti-ACE2 antibody (Abcam, Cambridge, UK, 15348, 1:100) in 10% (*v*/*v*) goat serum overnight at 4 °C. Next, samples were washed with 1x PBS (4 × 5 min) and incubated with secondary antibody (Alexa Fluor™ 488 goat anti-rabbit, 1:1000, Invitrogen, Carlsbad, CA, USA) and DAPI (Thermo Scientific, 1:500), at RT protected from light. Then, samples were washed 4X with 1X PBS and imaged using a Nikon AX/AX R confocal microscope system and analyzed by NIS Elements software (AR 5.02.01, Nikon, Melville, NY, USA).

### 2.3. Tissue Processing and Immunocytochemistry

Small intestine sample and salivary parotid and minor gland samples were placed in 10% *v*/*v* formalin for 24 h, paraffin embedded and sectioned (5 µm sections) by the Oral and Maxillofacial Pathology Core. Deparaffinization was conducted with Histo-Clear^®^ (Electron Microscopy Sciences, Hatfield, PA, USA, 64110) followed by rehydration in decreasing concentrations of ethanol. Three conditions were tested—no antigen retrieval, antigen retrieval with AR6 Buffer (Akoya Biosciences, Marlborough, MA, USA), or antigen retrieval with AR9 Buffer (Akoya Biosciences). Heat-mediated antigen retrieval steps were conducted in a Biogenex (Biogenex, Fremont, CA, USA) microwave at 95 °C for 15 min. Slides were cooled to room temperature and washed with 1X PBS. Sections were permeabilized with 0.3% Triton x-100 (*v*/*v*) for 30 min followed by a one-hour blocking step with 10% (*v*/*v*) normal goat serum and 0.3% Triton x-100. Subsequently, sections were incubated with primary antibodies overnight at 4 °C as follows: ACE2 monoclonal antibody (ProteinTech, Rosemont, IL, USA, 66699, 1:100), ACE2 monoclonal antibody (Abcam, 272500, 1:100), ACE2 polyclonal antibody (Abcam, 15348, 1:1000), Pan-cytokeratin (Abcam, AB234297, 1:100) or α-SMA (Abcam, AB7817, 1:100). Following the primary incubation, sections were washed 3X with PBS and incubated with the following secondary antibodies: goat anti-rabbit 488 (Invitrogen, A11008, 1:1000), goat anti-mouse 568 (Invitrogen, A11004, 1:1000), goat anti-rabbit 568 (Invitrogen, A11036, 1:1000), or goat anti-mouse 488 (Invitrogen, A11029, 1:1000). DAPI was used to visualize the nuclei (Thermo Scientific, 62247, 1:500 of 1mg/mL stock). A full list of antibodies used can be found in Table S1. Confocal fluorescence microscopy imaging was performed on the Nikon A1R/MP at the Center for Craniofacial Research Instrumentation Core, School of Dentistry, UTHealth Houston (Houston, TX, USA).

### 2.4. RNA Isolation and qPCR

Cells were lysed with 1ml of TRIzol^®^ LS Reagent (Life Technologies, Carlsbad, CA, USA, 10296028) and collected in Eppendorf tubes. After lysis, the Direct-zol RNA mini prep kit was used (Zymo Research, Irvine, CA, USA, R2052) according to the manufacturer’s protocol. Briefly, equal volumes of 100% ethanol were used, and each mixture was transferred to the spin column. Samples were spun and treated with DNAse 1. Washed samples were eluted with nuclease-free water. RNA purity and concentration were measured using a nanodrop (Thermo Scientific Nanodrop™ 2000,Thermofisher, Waltham, MA, USA). Following RNA isolation, a two-step PCR protocol was conducted. First, cDNA was generated using qScript cDNA SuperMix (Quantabio, Beverly, MA, USA, 95048-1000) according to the manufacturer’s protocol. Briefly, RNA (1 µg) was added to 4 µL of qScript cDNA; water was added to balance 20 µL. The cDNA was made in a thermocycler with the following parameters: incubate at 25 °C for 5 min, 42 °C for 60 min, then 85 °C for 5 min. After cDNA was constructed, the PCR reaction was conducted with the PerfeCTa SYBR^®^ Green SuperMix (Quantabio, 95053) with human ACE2 primers [32] forward 5′ TCCATTGGTCTTCTGTCACCCG 3′ and reverse 5′ AGACCATCCACCTCCACTTCTC 3′ and GAPDH primers 5′ TTGAGGTCAATGAAGGGGTC 3′ and reverse 5′ GAAGGTGAAGGTCGGAGTCA 3′. Relative gene expression was normalized to GAPDH expression, and CACO2 cells were used as a calibrator using the 2^−ΔΔCt^ method.

### 2.5. Protein Isolation and Western Blot

Cells were lysed with RIPA Buffer that included EDTA and protease and phosphatase inhibitors. A BCA assay was conducted and read on Biotek Cytation 5 (Agilent, Santa Clara, CA, USA) to determine protein concentrations and total protein (20 µg) was added. When using protein isolated from the overexpressing ACE2-HEK293T cell line, only 5 µg of total protein was added. Protein was added to Laemmli buffer and boiled at 100 °C for 5 min. Samples were electrophoresed on 10% Bis-Tris gels for 1–1.5 h with MOPS running buffer. Following the gel separation, wet transfer was carried out to transfer protein to a nitrocellulose membrane. The transfer was performed with 1X TG buffer at 50 V for 5 h with an ice block at 4 °C. Nitrocellulose membranes were washed 3X with 1X TBST buffer and blocked for one hour at room temperature with 3% BSA (Fraction V, heavy metal reduced). The following primary antibodies were incubated overnight at 4 °C: ACE2 monoclonal antibody (Abcam AB272500 1:1000) and GAPDH (Invitrogen, PA1-987, 1:10,000). After 3 washes, secondary goat anti-rabbit HRP (1:50,000) was administered for one hour. Finally, additional 3 washes were completed and then HRP chemiluminescent substrate was used; the blot was exposed to x-ray film, developed on a Konica Minolta SRX-101A (Konica Minolta, Whitsett, NC, USA), and then scanned on an Epson Perfection V30 (Epson, Hillsboro, OR, USA).

For whole tissue extracts, tissue was homogenized with a Dounce homogenizer (Wheaton, New Jersey, NJ, USA) on ice with RIPA buffer, EDTA, and protease/phosphatase inhibitors. Samples were spun at 10,000× *g* for 15 min, and the clear supernatant layer was extracted. Samples were aliquoted and stored at −20 °C until all collections were completed. The BSA assay for protein concentration and remaining Western blot protocol followed the cellular protocol except total protein loaded was 40 µg. The small intestine fresh-frozen positive control sample was purchased from BioIVT (Woodbury, NY, USA). The whole tissue blots were imaged on a ChemiDoc™ MP imaging system (Bio-Rad, Hercules, CA, USA).

### 2.6. Flow Cytometry

ACE2: Cells (salivary primary cells or ACE2-HEK293T) were trypsinized and resuspended in PBS with calcium and magnesium. Cells were prepared and then blocked with 10% goat serum in 1X PBS followed by incubation with the ACE2 monoclonal antibody (Ab272500, 1:500). Cells were washed three times with 1X PBS and the secondary antibody goat anti-rabbit 488 was used.

Spike Binding: Live/Dead Fixable Aqua was used to identify live cells (Invitrogen, L34965). Cells were untreated, treated with either Alpha spike protein (R&D Systems, Minneapolis, MN, USA, AFG10796) or Omicron BA.2 (R&D Systems, AFG11109) for 30 min followed by 3 washes with 1X PBS. Samples were kept on ice for the duration of the staining. Data were acquired on a BD Biosciences LSRFortessa™ (BD Biosciences, Franklin Lakes, NJ, USA) and analyzed with FlowJo software v10. Cells again were washed 3X and analyzed as described above.

### 2.7. Pseudoviral Infection

Full-length SARS-CoV-2 Spike Pseudovirus was generated using the Lenti-Pac™ SARS-CoV-2 Full-Length Spike protein-pseudotyped (D614G) Lentivirus Packaging Kit (Genecopoeia, LT016) according to manufacturing instructions. Briefly, the eGFP expression plasmid was expanded using NEB^®^ Stable Competent *E. coli* (Fisher Scientific, Waltham, MA, USA, NC0768018). Bacteria were thawed on ice and incubated with 4 µL of plasmid DNA (1 µg of plasmid) for two minutes. The *E. coli* was heat-shocked at 42 °C for 30 s and left to rest on ice for five minutes. NEB 10-beta/Stable Outgrowth media was added to the mixture and placed at 30 °C for one hour with rotation at 250 RPM. Bacteria were spread onto ampicillin (100 µg/mL) selection plates. Single colonies were selected and expanded in liquid LB cultures containing ampicillin at 30 °C overnight in a bacterial shaker set to 250 RPM (Thermo Scientific, MaxQ 4000). Liquid cultures were centrifuged and plasmid DNA was isolated and purified with the QIAprep Spin Miniprep Kit (Qiagen, Germantown, MD, USA, 27104). Plasmid concentration and purity was read using a nanodrop (Thermo Scientific, NanoDrop 2000).

Following expansion of the eGFP plasmid, 1 × 10^6^ HEK293T (ATCC, CRL-3216) packaging cells were plated into a 10 cm dish in 10 mL of DMEM supplemented with 10% (*v*/*v*) fetal bovine serum (FBS). Two days later, when cells reached 70–80% confluency, 2.5 µg of eGFP plasmid and 2.5 µg of the Lenti-Pac full-length spike plasmid mix were added to 200 µL of Optimem. Separately 15 µL of EndoFectin was added to 200 µL of Opti-MEM. Endofectin mixture was added dropwise to plasmid DNA tube and left at room temperature for 25 min. The DNA–Endofectin complex was added to the HEK293T packaging cells dropwise. The next day, the media was replaced with fresh DMEM supplemented with 4% FBS and 1/500 volume of the supplied TiterBoost reagent. Then, 48 h post-transfection, media was collected and centrifuged at 500× *g* for 10 min to remove cell debris. The collected supernatant was layered onto a sucrose cushion containing 10% sucrose (*w*/*v*), 50 mM Tris-HCl, 100 mM NaCl, 0.5 mM EDTA, pH 7.4 and ultracentrifuged (Beckman Coulter, Brea, CA, USA, Optima L-100 XP), at 25,000 RPM for 2 h in a SW41Ti swinging-bucket rotor. Following centrifugation, the supernatant was discarded, and the viral cell pellet was reconstituted in PBS containing 1 mg/mL human serum albumin. Virus was aliquoted and stored at −80 °C until used for infection.

For infection studies, cultured basal cells from parotid salivary tissues denoted F22 and M88C (2 × 10^4^ cells/well), HEK 293T and ACE2-HEK 293T (1 × 10^4^ cells/well) were seeded into 96 wells with complete growth media and incubated for 48 h at 37 °C. SARS-CoV-2 pseudovirus was added to a mixture of complete salivary media or reduced serum media (5% *v*/*v* FBS) for HEK 293T and ACE2-HEK 293T with ViralEntry™ Transduction Enhancer (100X, G515, 1:100, Applied Biological Materials Inc., Richmond, BC, Canada) or Polybrene Transfection Reagent (TR-1003, Thermo Fisher) at final concentration of 5 μg/mL, and mixtures were added to cells and incubated at 37 °C. A mock-uninfected cell group was added as control. Then, 72h post-infection, confocal microscopy (Nikon AX/AX R Confocal Microscope System, Nikon, Melville, NY, USA) was used to observe eGFP fluorescence associated with viral entry. Statistical analysis one-way ANOVA was used to analyze GFP expression. Additional details are provided in the figure legends. For this work, *p*-values < 0.05 were considered statistically significant. GraphPad Prism software (version 10.2.3; GraphPad Software, La Jolla, CA, USA) was used for data analysis.

## 3. Results

### 3.1. Tissue-Level Examination of ACE2 in Minor Salivary Glands and Parotid Tissue

In the first set of studies using fluorescence-based immunocytochemistry on fixed tissue, we tested a series of three antibodies that have been used in various laboratories for detection of ACE2 protein in fixed tissues from both major (parotid) and minor salivary gland tissues. Small intestinal tissue served as the positive control. Because previous studies have inconsistently reported presence or absence of ACE2 in salivary tissues, we systematically compared the signal obtained without antigen retrieval and after antigen retrieval at pH 6 and pH 9. In each case, tissues were counterstained with either α-smooth muscle actin (α-SMA) or a pan-cytokeratin (pan CK). In salivary tissues, α-SMA serves as an excellent marker for myoepithelial cells surrounding each salivary acinus, while pan CK should stain all epithelial cells.

**Figure 1:**
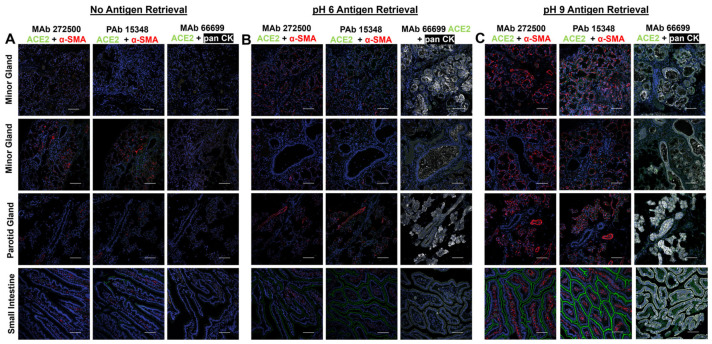
Immunofluorescence labeling in salivary glands and small intestine tissue sections shows lack of angiotensin-converting enzyme 2 (ACE2) in major and minor salivary glands. Three anti-ACE2 antibodies each tested under three retrieval conditions were used for detection of ACE2 with small intestine serving as the positive control. (**A**) ACE2 immunodetection without antigen retrieval. (**B**) ACE2 immunodetection after pH 6 antigen retrieval (**C**) ACE2 immunodetection after pH 9 antigen retrieval. In all panels ACE2 is labeled green and α-SMA red or pan-CK white. DAPI is used to visualize nuclei in blue. Note that the images of tissues are from serial sections, hence the morphological structures seen in the sections appear similar. scale bar 100 µm.

Figure 1 shows the results of the immunocytochemical analysis. Each group (no antigen retrieval, pH 9, and pH 6) includes thin 5 µM serial sections, chosen to maximize tissue composition similarity among images within a specific immunocytochemistry group. Without antigen retrieval (Figure 1A panels, bottom row), no ACE2 labeling was observed in the small intestine positive control. This was the case for all three ACE2 antibodies, one rabbit polyclonal (PAb15348) and two mouse monoclonals (MAb272500, MAb66699) (Figure 1A). Likewise, without retrieval, no ACE2 was detected in parotid gland nor two minor salivary gland specimens (Figure 1A, top three rows). In the small intestine not subjected to antigen retrieval, α-SMA showed scattered and discontinuous staining in the lamina propria (red color) with two of the antibodies used, and pan-CK was not observed. Modest, diffuse staining for α-SMA was seen in the acinar, but not ductal, regions of both parotid and minor salivary glands. Given these negative findings, all subsequent studies used antigen retrieval.

In the studies shown in Figure 1B, all fixed, serial sections underwent antigen retrieval with a pH 6-based antigen retrieval, AR6 buffer. After retrieval, the polyclonal antibody (Mab15348)-treated specimens showed robust signal on the apical side of the small intestine epithelium (Figure 1B, bottom row). Similarly, the monoclonal antibody (ab272500)-treated small intestine sections showed fainter, but still positive, signal in the same location. Sections treated with the second monoclonal antibody (MAb66699) showed little or no positive signal in the small intestine even after pH 6 antigen retrieval. After retrieval at pH 6, α-SMA was visible in the lamina propria of the small intestine and pan-CK (white) had a slightly positive signal in the epithelial compartment. For the salivary gland sections examined (Figure 1B, top three rows), no staining for ACE2 was seen with any of the three anti-ACE2 antibodies. Signal for α-SMA was observed in both acinar and vascular cells with robust staining of both acinar and ductal epithelial cells seen with pan-CK.

To determine if a pH 9-based antigen retrieval would yield clearer results, a pH 9, AR9 buffer was used. After this treatment, both MAb272500 and PAb15348 antibodies for ACE2 showed strong and robust signal (green) on the apical surface of the small intestinal epithelium (Figure 1C, bottom row). Detection for α-SMA showed strong signal localized to the lamina propria compartments of the small intestine not overlapping the staining for ACE2. Anti-CK staining of the epithelial compartment was also strong and uniform after retrieval at pH 9. Examination of salivary gland sections after retrieval at pH 9 (Figure 1C, upper three rows) showed strong staining for α-SMA in the myoepithelial component of both salivary parotid and minor glands, as well as vascular elements. No staining of salivary ducts was observed. Pan-CK staining showed strong expression in the epithelial compartment including both acinar and ductal cells present in parotid and minor salivary glands. In contrast, even under conditions where all three anti-ACE2 antibodies produced strong signal in positive control, no ACE2 staining was visible in either the parotid or minor salivary glands. The signal was absent in both acinar and ductal compartments, even when these distinct structures were clearly visible in the specimens.

Thus, from viewing results of this systematic immunostaining panel, it became clear from this study of ACE2 protein expression in both major and minor salivary glands from multiple individual donors, using three different validated antibodies shown to work in other tissues and three different conditions for immunostaining, that there is likely to be very little ACE2 present on the surfaces of exocrine salivary cells.

**Figure 2:**
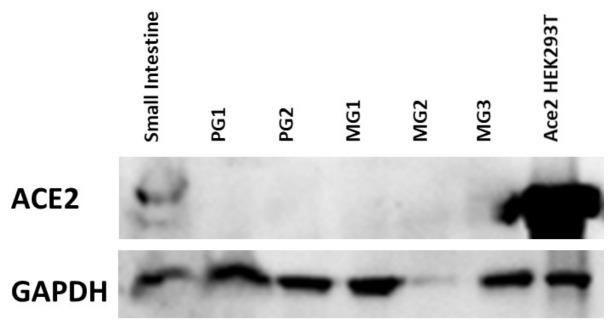
ACE2 detection in protein extracts from small intestine, major and minor salivary glands and ACE2-HEK293T cells. Western blotting for ACE2 (**upper bands**) and GAPDH (**lower bands**) in small intestine (left), and five salivary glands from different patient donors, including two parotid and three minor salivary glands. ACE2-HEK293T cells are shown on the right and were used as an additional positive control along with small intestine. No signal was seen in any salivary tissue extract even after prolonged exposure.

To verify if this lack of ACE2 protein in salivary glands was true using an alternative method, whole tissue lysates were analyzed by Western blot. As shown in Figure 2, no positive signal for ACE2 was seen in protein extracted from two parotid glands and three minor glands even when overexposed. In contrast, positive signal was seen for both small intestine lysate and a cell line, ACE2-HEK293T, constitutively producing ACE2 from a stably expressed transgene.

### 3.2. Cellular Examination of ACE2 Transcripts and Protein in Salivary Basal Cell-Dervived hS/PCs

**Figure 3:**
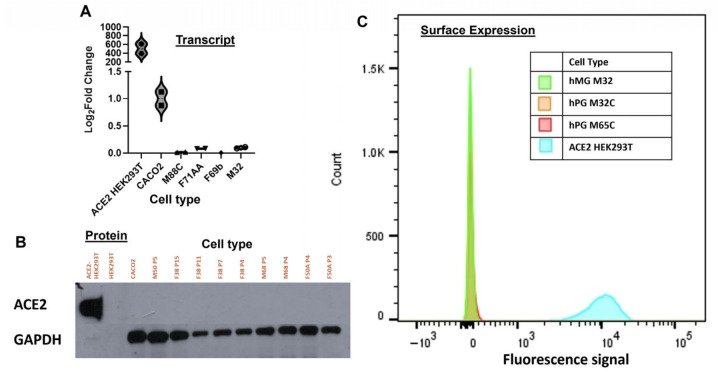
Lack of detectable ACE2 transcript, protein or cell surface ACE2 expression in salivary basal cells. Three methods of detection were used to examine ACE2 levels in isolated, cultured hS/PCs equivalent to salivary basal cells. (**A**) qPCR results showing Log_2_Fold Change normalized to CACO2 intestinal cells. Left to right are amplimers from the ACE2-HEK293T cell line, CACO2, and four different patient-derived salivary basal cell cultures. (**B**) Western blot results from protein extracts from four patient-derived basal cell cultures from major and minor glands and grown to various passages. Left lane is ACE2-HEK293T cell extract followed by CACO2 and then patient-derived salivary basal cell extracts ranging from early passage (3) to late passage (15). (**C**) Flow cytometry analysis for ACE2 present on the surfaces of living cells. ACE2-HEK293T is shown in cyan; three salivary basal cell lines, two parotid gland-derived are shown in orange and red, and one minor salivary gland-derived is shown in green.

Given our inability to identify ACE2 protein in salivary tissue by immunocytochemistry under the best retrieval conditions, we next sought to determine using various techniques if ACE2 transcripts or protein were present in expanded cultures of pluripotent, primary salivary epithelial cells. Salivary cells derived from minor and major glands of multiple patients were examined using qPCR-based methods and validated primers specific for human ACE2 transcripts. ACE2-HEK293T cells served as positive control and expressed high levels of transcript, ~500-fold increase over CACO2 cells (Figure 3A). CACO2 cells also expressed ACE2 transcripts, but at significantly lower levels than ACE2- HEK293T’s (Figure 3A). Four salivary primary cell populations from patients of different sexes and backgrounds were tested independently (M88C, F71AA, F69b, and M32). None of these cultured lines from major or minor (M32) glands were found to express detectable levels of ACE2 transcripts (Figure 3A). Considering that expression of ACE2 by salivary cells might be a result of chronic inflammation during SARS-CoV-2 infection, we next set out to determine if we could force ACE2 expression through exposure to the inflammatory cytokines TNFα, IFNγ, or a combination of the two. After 24 h and 72 h of treatment, none of the treatments induced expression of ACE2 transcripts (Supplementary Figure S1).

To examine the potential presence of ACE2 at the protein level in salivary cells, a Western blot was performed using lysates from ACE2-HEK293T (positive control), HEK293T cells (negative control), CACO2 cells and expanded cultures of salivary cells from four patient donors across multiple passages to determine if ACE2 may be present in earlier passages and subsequently lost in later passages (Figure 3B). Of all lines tested, only the ACE2-HEK293T cells showed a strong positive band. Even the CACO2 cells were negative, suggesting low levels of ACE2 protein in this cell line. ACE2 was undetectable in all patient-derived salivary cells, again representing patients over a wide range of ages, different sexes and various backgrounds. Additionally, passage number did not affect the lack of expression of ACE2 protein, which was undetectable in both low (1–4) and high passage (8–12) cells.

To further examine the absence of ACE2 protein on cell surfaces that might be at levels lower than could be readily detected by Western blotting and to avoid the possibility that these assays were not sensitive enough to detect low levels of surface ACE2, we next conducted flow cytometry for ACE2 in primary cultured salivary cells from three individual patient donors. Again, salivary cells showed a total absence of fluorescence intensity in contrast to the positive control ACE2-HEK293T cells (Figure 3C). Taken together, these results indicated that ACE2 transcripts and protein are not detectable in primary cultures of salivary epithelial cells.

### 3.3. Spike-Binding Assay on Salivary Basal Cells

**Figure 4:**
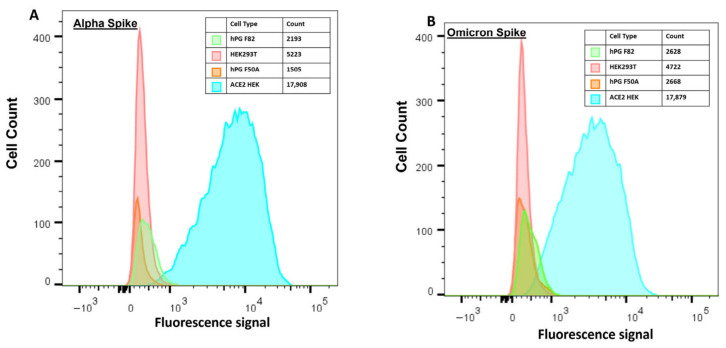
Flow cytometry to detect binding of fluorescently labeled spike proteins corresponding to alpha and omicron spike variants in living salivary cells. Two variants of spike protein were prepared as described in Methods and assessed for cell surface binding. (**A**) Fluorescence intensity histogram with fluorescently labeled alpha spike protein bound to ACE2-HEK293T’s (cyan, positive control). No cell surface binding was seen for the negative control HEK293Ts (red) nor to two parotid patient-derived basal cell populations (orange and green). (**B**) Fluorescence intensity histogram with fluorescently labeled omicron spike protein bound to ACE2-HEK293T’s (cyan). No surface binding was seen for HEK293Ts (red) or for two parotid patient-derived basal cell populations (orange and green).

Given the surprising lack of ACE2 seen in salivary tissue or cells, we sought to determine if there could be an alternative receptor on salivary cells that could bind SARS-CoV-2 spike protein to allow infection. Use of spike protein, unlike an antibody, offers an agnostic means to test the ability of the virus to bind to living cell surfaces. To verify if there was an ACE2-independent mechanism by which spike protein could bind to the surfaces of salivary cells in the absence of ACE2, we conducted flow cytometry using fluorescently labeled spike proteins corresponding to an early circulating variant of interest, alpha, and a later circulating variant of interest, omicron. We tested both spike proteins on our salivary cell populations from two patients, ACE2-HEK293T (positive control) and parental HEK293T (negative control). Flow cytometry data indicated a shift in mean fluorescence intensity on binding of spike to ACE2-HEK293T cells for both the alpha spike protein variant (Figure 4A) and the omicron spike protein variant, indicating that the spike proteins bound to cells expressing ACE2, as expected (Figure 4B). The lack of fluorescent shift indicated that basal cells derived from parotid gland were unable to bind either variant along with the unmodified HEK293T cell line. Additional controls are provided in Supplementary Figure S2.

Finally, we sought to test spike binding using whole tissue sections. Formalin-fixed sections and fresh-frozen sections did not show measurable GFP signal (Supplementary Figure S3), unsurprising since spike binding is thought to require a conformational change not possible in fixed tissue.

### 3.4. SARS-COV-2 Pseudovirus Entry into Cultured Salivary Basal Cells

**Figure 5:**
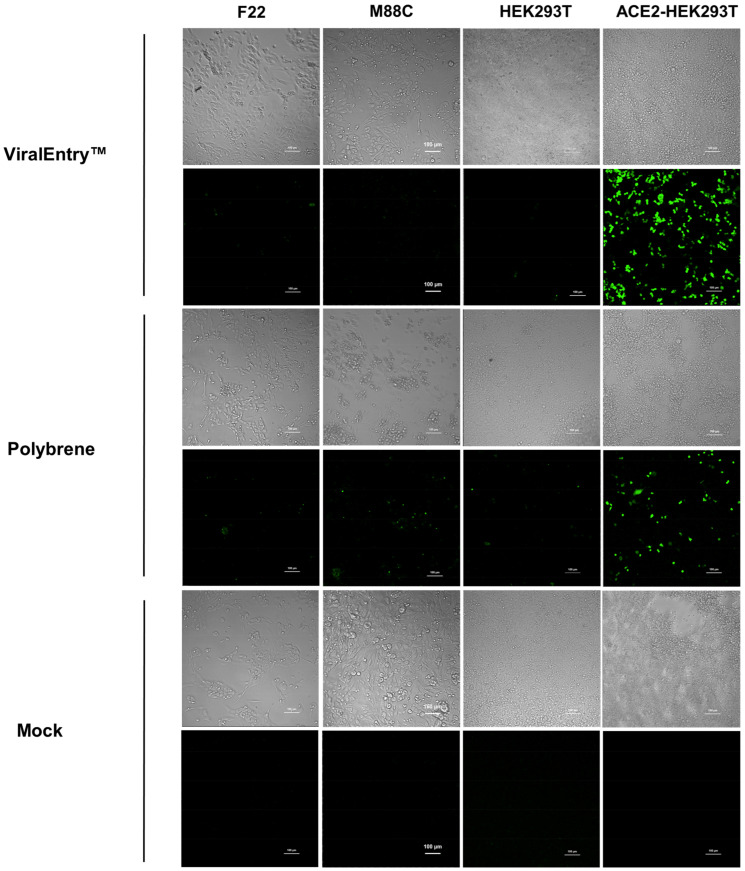
Testing SARS-CoV-2 pseudovirus entry into hS/PCs and control cell lines. Human salivary cells derived from parotid tissues (F22 and M88C; 2 × 10^4^ cells/well), HEK293T, and ACE2-expressing HEK293T cells (1 × 10^4^ cells/well) were seeded into 96-well plates and cultured for 48 h at 37 °C in complete growth media. Cells were exposed to SARS-CoV-2 pseudovirus prepared in either complete salivary medium or reduced-serum medium (5% *v*/*v* FBS; HEK293T and ACE2-HEK293T), supplemented with ViralEntry™ Transduction Enhancer (1:100; Applied Biological Materials Inc.) or Polybrene (5 µg/mL; Thermo Fisher Scientific). Mock-treated wells served as uninfected controls. After 72 h, viral entry was assessed by confocal microscopy via detection of eGFP reporter expression. Representative phase-contrast (**top panels**) and corresponding fluorescence images (green; eGFP, **lower panels**) are shown for each condition. Minimal eGFP signal was observed in parental HEK293T and salivary cultures, whereas robust fluorescence was detected in ACE2-HEK293T cells, confirming receptor-dependent viral entry.

To determine if human salivary cells lacking ACE2 are permissive to SARS-CoV-2 entry, we compared pseudoviral transduction across two primary salivary cell isolates (F22, M88C), parental HEK293T cells, and ACE2-expressing HEK293T controls. Infection was assessed by eGFP reporter expression following exposure to pseudovirus under conditions optimized with either ViralEntry™ enhancer or Polybrene. Across both salivary isolates and parental HEK293T cells, eGFP signal remained at or near background levels, with only rare, punctate fluorescence observed (Figure 5). In contrast, ACE2-HEK293T cells exhibited a marked increase in eGFP-positive cells, with widespread and robust fluorescence indicative of efficient viral entry (Figure 5). This pattern was consistent regardless of the transduction enhancer used, indicating that enhancement of viral uptake did not overcome the absence of ACE2. Quantitative analysis confirmed a significant increase in eGFP signal exclusively in ACE2-expressing cells compared to all other groups (one-way ANOVA, *p* < 0.05), while no significant differences were detected among salivary cells, parental HEK293T, or mock-treated controls. Staining for ACE2 by immunocytochemistry for all cells used in this experiment is shown in Supplementary Figure S4. Together, these data demonstrate that entry of the SARS-CoV-2 pseudovirus is dependent on ACE2 expression and that primary human salivary cells, under these conditions, do not support appreciable viral entry.

## 4. Discussion

Our investigation into the presence and potential involvement of ACE2 in salivary gland tissues and basal-derived salivary epithelial cells revealed a consistent lack of both transcript and protein, despite a methodologically rigorous approach that included multiple detection techniques and antibody optimization conditions. The lack of ACE2 RNA transcripts and protein levels in basal cells derived from both parotid and minor salivary glands (Figure 1) was surprising given that the literature indicates that basal ductal cells were likely to have ACE2 [24,33]. These findings directly contradict some earlier studies and suggest that salivary glands are unlikely to serve as major entry points or reservoirs for SARS-CoV-2 via an ACE2-dependent route.

Our first studies evaluated ACE2 protein expression at the tissue level in both parotid and minor salivary glands using immunocytochemistry. To address variability seen in the literature, we systematically tested three well-characterized ACE2 antibodies under a range of antigen retrieval conditions (no retrieval, pH 6, and pH 9) including those antibodies and conditions reported in the cited literature. Our positive control, small intestinal tissue, exhibited robust apical ACE2 staining under pH 6 and pH 9 retrieval conditions, confirming the efficacy of the antibodies and the staining protocol. In contrast, salivary tissues showed no ACE2 signal under any condition, despite the clear labeling of epithelial (pan-cytokeratin) and myoepithelial (α-SMA) cells. These results indicate that neither acinar nor ductal compartments of the parotid and minor salivary glands express detectable ACE2 protein.

To validate these findings with a complementary approach, we performed Western blotting on lysates from both parotid and minor salivary glands. Consistent with the immunostaining data, ACE2 protein was undetectable in all salivary gland samples, whereas it was readily observed in small intestine lysates and ACE2-overexpressing HEK293T cells. These results support the conclusion that ACE2 is not expressed at significant levels in exocrine salivary cells under basal conditions. A limitation of this work is that the tissues and cells we analyzed were normal tissues from patients undergoing surgery for other reasons without active infection or signs of salivary inflammation. It is possible that ACE2 is absent in normal salivary tissues, but induced in conditions of inflammation or injury, a possibility that would be worthy of future study. We note, however, that we were unable to induce expression of ACE2 in our salivary cells with various inflammatory cytokines (Supplementary Figure S1).

Given the potential for disease-induced upregulation or the presence of receptor expression below detection thresholds in tissue sections, we extended our investigation to primary human salivary epithelial cell cultures (hS/PCs) derived from both major and minor glands of multiple patients across sex, age, and ethnic backgrounds. Quantitative RT-PCR showed that none of these cell lines expressed detectable ACE2 transcripts, even after treatment with the pro-inflammatory cytokines TNFα and IFNγ for 24 and 72 h. Furthermore, Western blot analysis of four different donor-derived cell cultures across multiple passages again showed no ACE2 protein, and this was confirmed with highly sensitive flow cytometry. Across all patient-derived lines, there was no detectable ACE2 surface expression, while ACE2-overexpressing HEK293T cells consistently served as a strong positive control.

While ACE2 is the primary receptor for SARS-CoV-2, there have been several publications that have implicated additional receptors for the virus to bind and enter through integrins [26,34]. To explore the possibility of ACE2-independent viral binding mechanisms, we used spike proteins corresponding to two SARS-CoV-2 variants (alpha and omicron) labeled for fluorescence to probe basal salivary cells via flow cytometry. This method provides a direct, receptor-agnostic approach to assess potential viral engagement with the cell surface. However, no binding was observed in salivary cells from any patient donor, in contrast to the strong fluorescent shifts seen in ACE2-expressing HEK293T cells. These data indicate that neither canonical ACE2-dependent nor alternative receptor-mediated spike binding occurs in basal salivary epithelial cells under the conditions tested. This idea is further supported by our inability to directly infect primary salivary cells using a fluorescently labeled pseudovirus, while ACE2-positive cells were readily infected. A limitation of the work we present here is that we used a pseudotyped lentivirus (D614G spike) to assess viral entry. Because pseudovirus assays are only able to measure entry, they cannot convey further information about potential for replication, viral release, or any cytopathic effect. Additionally, the D614G variant may not represent the entry potential of recent omicron sub-lineages. Nonetheless, the absence of ACE2 in salivary cells indicates that any entry pathway for these sub-lineages would most likely involve receptors unrelated to ACE2.

These findings have significant implications for understanding viral interactions in the oral cavity. Early in the COVID-19 pandemic, public transcriptomic databases and single-cell RNA sequencing data suggested the salivary glands might express ACE2, leading to speculation that ducts could serve as viral portals or reservoirs [24,35,36]. However, those datasets showed only low, cell-type-specific ACE2 mRNA expression, and many lacked robust validation at the protein level. Our data indicate that even under optimized conditions, salivary epithelial cells lack both transcript and protein expression of ACE2. While some papers have shown ACE2 protein in the ducts [37] or both acinar cells and ducts [38,39], the literature routinely shows inconsistency in immunocytochemistry results as well as a robust protein expression that does not correlate with the transcript levels. Additional studies supported our results suggesting that the salivary glands do not have ACE2 at the protein level [7] and another study suggested that the inconsistency between these data may be a result of the overlap in homology between ACE2 collectrin-like binding domain [40]. The inconsistency across the literature may partly stem from the use of antibodies with cross-reactivity to domains with high homology to the ACE2 such as collectrin, potentially leading to false-positive immunostaining results.

Considering possible retrograde paths of entry for SARS-CoV2 through ducts open in the oral cavity, it should be considered that humans possess three pairs of major salivary glands, parotid, submandibular and sublingual [18,41]. The largest gland, the parotid, is connected to the oral cavity through Stensen’s duct, typically 5–6 cm in length and 1–3 mm in diameter. The gland opens opposite the second maxillary molar, via a papilla in the buccal mucosa, and opens into the oral cavity. The parotid gland is serous, producing copious amounts of watery, amylase-rich saliva. The submandibular gland is connected to the oral cavity through Wharton’s duct, approximately 5 cm in length and 1–3 mm in diameter and produces a mixed saliva that is the product of both serous and mucous acinar cells. Wharton’s duct opens at the sublingual caruncle, under the tongue. The sublingual gland is the most mucous-rich of the major glands and does not rely on a single large duct like the parotid and submandibular glands. Rather, 8–20 small, shorter ducts of Rivinus (1–2 mm) open directly onto the floor of the mouth alongside the sublingual fold. Bartholin’s duct (1–2 cm in length), when present, may join Wharton’s duct or have a nearby separate opening. Unstimulated saliva in normal salivary glands flows at rates of ~0.3–0.5 mL/min, whereas stimulated flow reaches rates of ~1.5–2.0 mL/min, with daily production reaching up to 1.5 L per day [42,43]. SARS-CoV-2 virus particles present in the oral cavity would need to move against a significant flow of fluid, then penetrate the mucous-lined walls of the ducts [44], to enter and infect the secretory acinar component of the major salivary glands.

**Figure 6:**
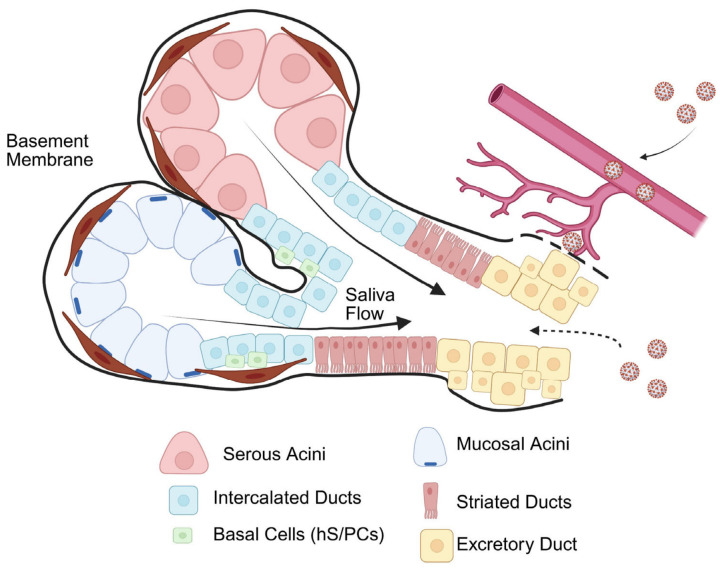
Schematic showing possible routes of major salivary gland infection by SARS-CoV-2. Saliva flow is unidirectional after secretion from the serous and mucous acini. After secretion, whole saliva is transported through the ductal network before entering the oral cavity. ACE2-dependent viral infection of major ducts through ACE2, if present on the surfaces of salivary ducts, would require retrograde ductal passage of virus from the oral cavity to reach surface receptors. The lack of detectable ACE2 suggests this is not a major route of infection. Basal cell infection would require transepithelial invasion of virus or encroachment across the basement membrane (solid and dotted black line). Salivary glands are highly vascularized; systemic SARS-CoV-2 viremia and inflammation could compromise the integrity of the basement membrane (dotted black line) allowing the virus to travel from the vasculature to the salivary gland where infection could be established in the stem-/progenitor-like basal cells through an ACE2-independent route. The lack of spike binding by salivary basal cells indicates that this is not a likely alternate path of infection (created in BioRender. Farach-Carson, M. (2026) https://BioRender.com/rpn7ydv, accessed on 30 April 2026).

Moreover, our findings argue against models proposing retrograde infection of the salivary ducts from the oral cavity as a significant route of SARS-CoV-2 entry (Figure 6). If such a mechanism were valid, one would expect to observe ACE2 on ductal surfaces or successful spike protein binding in vitro, neither of which was observed in our study.

While our results do not support local epithelial susceptibility to SARS-CoV-2 via ACE2 in the salivary glands, we acknowledge that glandular involvement during systemic viral infection remains plausible through hematogenous spread. Such systemic infections could have arisen through the following classical sites of entry: lung, gastrointestinal system, or the nasopharynx. Salivary glands are highly vascularized, and several viruses, such as mumps and CMV [45], can infect glandular tissues via the bloodstream [46,47]. In such cases, the infection may involve infected perivascular, immune or stromal cells rather than epithelial compartments. SARS-CoV-2 may similarly affect salivary tissues through systemic routes, potentially explaining the detection of viral RNA in salivary secretions in some patients [24], albeit without clear evidence of productive infection in glandular epithelial cells.

In summary, our systematic study clearly demonstrates that ACE2 is absent in basal cells and whole tissue from both major and minor salivary glands, regardless of tissue source (age, sex) or in the case of cultured cells, passage number. Further, these cells are not likely to be susceptible to initial infection of SARS-CoV-2 via spike binding, even through alternative pathways. These results collectively challenge prior assumptions regarding the role of salivary glands in direct SARS-CoV-2 pathogenesis and highlight the importance of multimodal validation in receptor expression studies of infection.

## 5. Conclusions

In conclusion, our data, together with the anatomical context of major and minor salivary gland architecture and position, support the hypothesis that direct infection of salivary glands via the oral cavity is a highly improbable first route of infection. The physical barriers posed by the mucosal layer, the protective properties of saliva, and the complexity of the ductal system, including Stensen’s and Wharton’s ducts, make retrograde viral entry and surface infection via ACE2 an unlikely route. Instead, we propose that in some cases, SARS-CoV-2 may reach the salivary glands through systemic dissemination, gaining access via the glandular vasculature, immune cells or stroma. This idea is consistent with the expression patterns and levels of ACE2 in cells and tissues and may help explain observed viral presence in glandular tissues including salivary glands of infected patients. Although the potential for spike-independent mechanisms of entry cannot be excluded, especially in cellular contexts where ACE2 is minimally expressed or absent, our infectivity data suggest that SARS-CoV-2 is unlikely to directly infect salivary cells via direct viral entry. Alternate pathways, possibly involving endosomal uptake or alternative receptors, may expand the tissue distribution of SARS-CoV-2 and warrant further investigation in glandular models. These insights contribute to a broader understanding of viral pathogenesis within the oral cavity and underscore the importance of considering local cellular susceptibility in determining infectivity.

## Data Availability

Data available upon reasonable request.

## Supplementary Materials

The following supporting information can be downloaded at https://www.mdpi.com/article/10.3390/biology15100778/s1; Table S1. Antibodies Used in this Study. Figure S1. Cytokine treatment fails to induce ACE2 protein expression in salivary cells as assessed by flow cytometry. Cells from patient donors were treated with inflammatory cytokines for one or three days, harvested and analyzed for ACE2 cell surface expression by flow cytometry as described in the Methods. Treatment for 24 h (top panels); Treatment for 72 h (bottom panels). Left to right: No treatment, TNF-α (10 ng/mL), IFN-γ (1000 Units/mL), combination treatment at same concentration of each. Inflammatory cytokines were replaced daily during the 72-h treatment. Figure S2. Unstained flow cytometry controls. Cells were treated with live/dead fixable aqua. Fluorescence intensity in the GFP channel was plotted as a histogram. Single fluorescence channel controls were performed for all four cell populations used in the spike-binding assay. No differences were observed. Figure S3. Fluorescently labeled spike protein fails to bind to bind specifically to fresh-frozen small intestine or 10% formalin fixed small intestine (the positive control for these studies). Fixed and fresh-frozen sections were incubated with either GFP-labeled Alpha Spike (left), GFP-labeled Omicron Spike (middle) or PBS negative control (right) overnight with DAPI. Diffuse green nonspecific signal background was seen at high laser power without spike addition in PBS control formalin fixed section (upper right panel) and in Omicron spike (upper middle panel). No specific signal was seen in any fresh frozen tissues examined (lower panels). This indicates a lack of spike binding in either 10% formalin (top) or fresh frozen (bottom) tissue sections. This contrasts with robust staining for ACE2 in these sections shown in Figure 1 in the main article. Figure S4. ACE2 expression is minimal/absent in salivary epithelial cell preparations. Representative immunofluorescence images of salivary gland cell cultures from two patient specimens. Nuclei are labeled with DAPI (blue) and ACE2 immunoreactivity is shown in green. Across all conditions examined, salivary epithelial cells exhibit little to no detectable ACE2 signal, with only rare, sparse puncta observed at background levels. In contrast, bright green signal (right panels) confirm robust staining in ACE2-HEKT cells. Merged images (bottom row) further demonstrate the absence of significant ACE2 co-localization with salivary epithelial cells. Scale bars as indicated. These data indicate that salivary gland epithelial cells in this system do not express appreciable levels of ACE2 protein under the conditions tested.

## Abbreviations

The following abbreviations are used in this manuscript:

SARS-CoV-2	Severe Acute Respiratory Syndrome Coronavirus 2
ACE2	Angiotensin-converting Enzyme 2
hS/PCs	Human stem/progenitor cells
α-SMA	α-smooth muscle actin
Pan CK	Pan-cytokeratin
