# Supplementary material for: Exploring Spike-Dependent and ACE2-Independent SARS-CoV-2 Interactions with Salivary Epithelial Cells in the Absence of ACE2
Source: Biology (Basel). 2026 May 14;15(10):778. doi: 10.3390/biology15100778 (PMC13203730; doi:10.3390/biology15100778)
Supplement: Supplementary file 1 [file biology-15-00778-s001.zip › biology-4267834-supplementary/biology-4267834-supplementary.pdf]

Supplementary Table S1. Antibodies Used in this Study

Antibody	Target	Description	Source
MAb 66699	ACE2	mouse monoclonal	Protein Tech
MAb 272500	ACE2	rabbit monoclonal	Abcam
PAb 15348	ACE2	rabbit polyclonal	Abcam
MAb7817	SMA	mouse monoclonal	Abcam
MAb234297	Pan-cytokeratin	rabbit monoclonal	Abcam
A11008	anti-rabbit	goat polyclonal (488)	Invitrogen
A11004	anti-mouse	goat polyclonal (568)	Invitrogen
A11029	anti-mouse	goat polyclonal (488)	Invitrogen
A11036	anti-rabbit	goat polyclonal (568)	Invitrogen

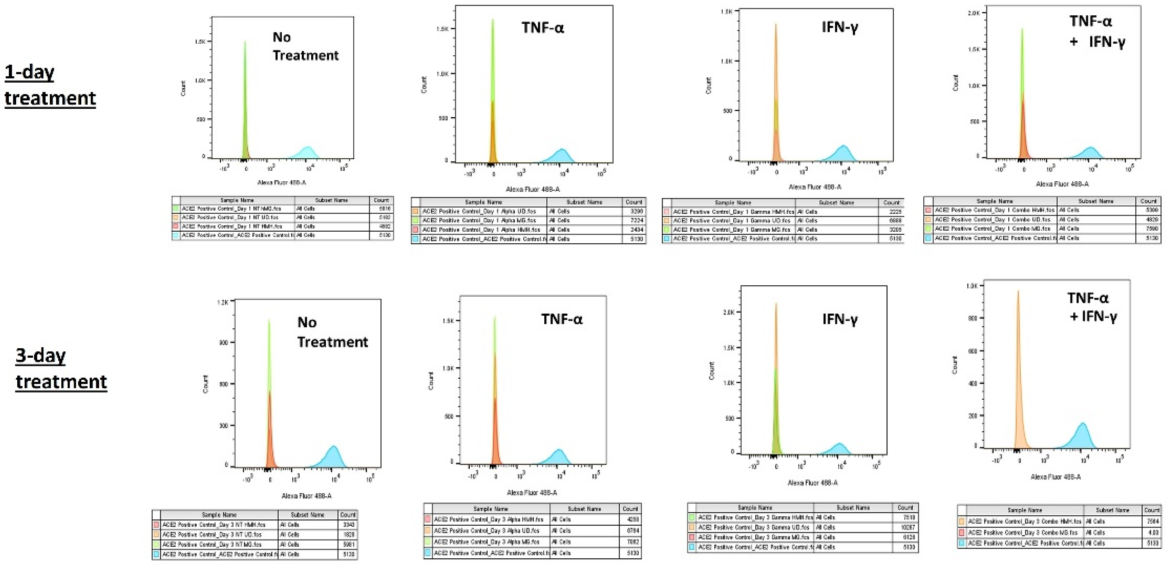

Supplementary Figure S1. Cytokine treatment fails to induce ACE2 protein expression in salivary cells as assessed by flow cytometry. Cells from patient donors were treated with inflammatory cytokines for one or three days, harvested and analyzed for ACE2 cell surface expression by flow cytometry as described in the Methods. Treatment for 24 h (top panels); Treatment for 72 h (bottom panels). Left to right: No treatment, TNF-α (10 ng/mL), IFN-γ (1000 Units/mL), combination treatment at same concentration of each. Inflammatory cytokines were replaced daily during the 72-h treatment.

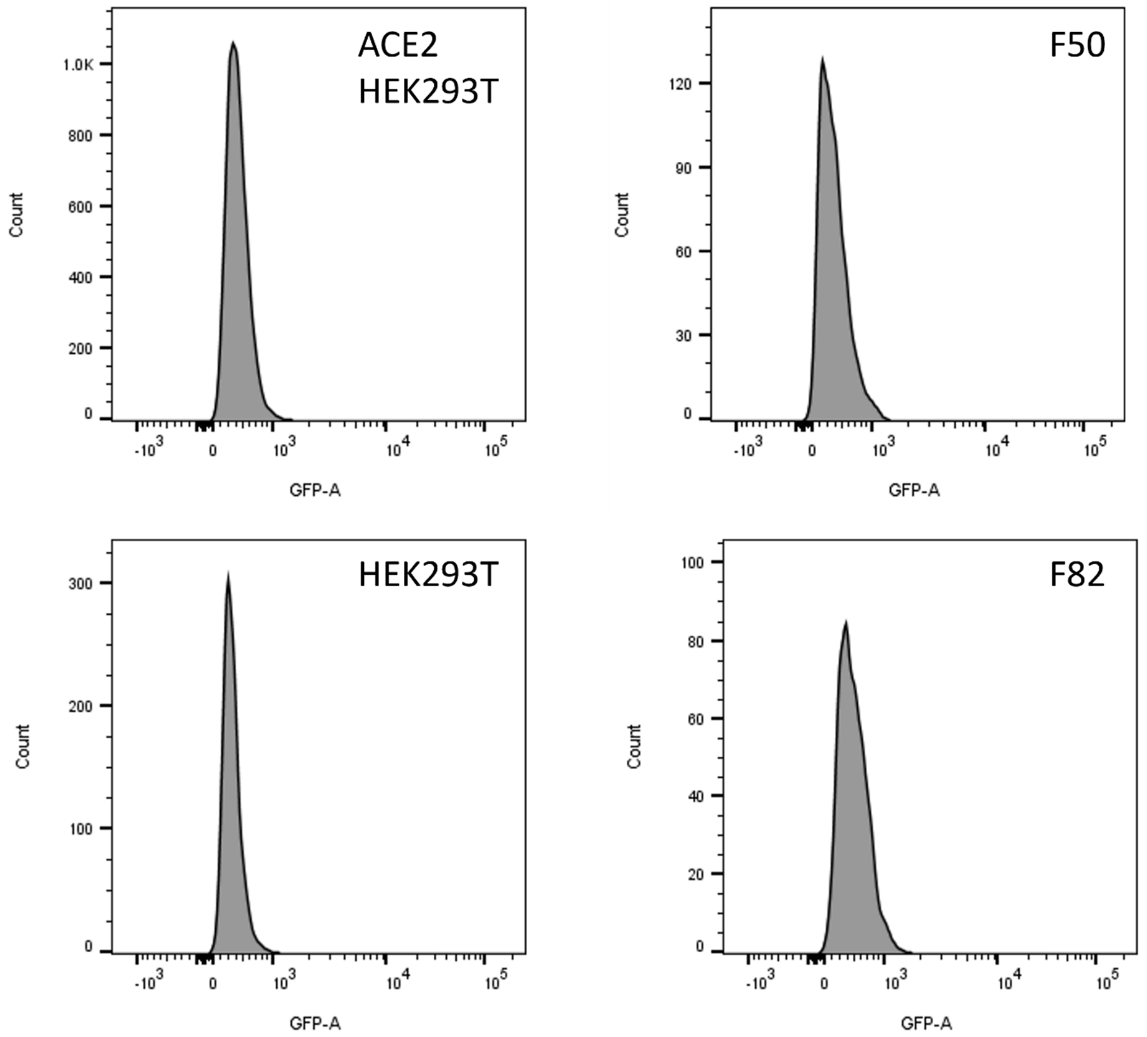

Supplementary Figure S2. Unstained flow cytometry controls. Cells were treated with live/dead fixable aqua. Fluorescence intensity in the GFP channel was plotted as a histogram. Single fluorescence channel controls were performed for all four cell populations used in the spike-binding assay. No differences were observed.

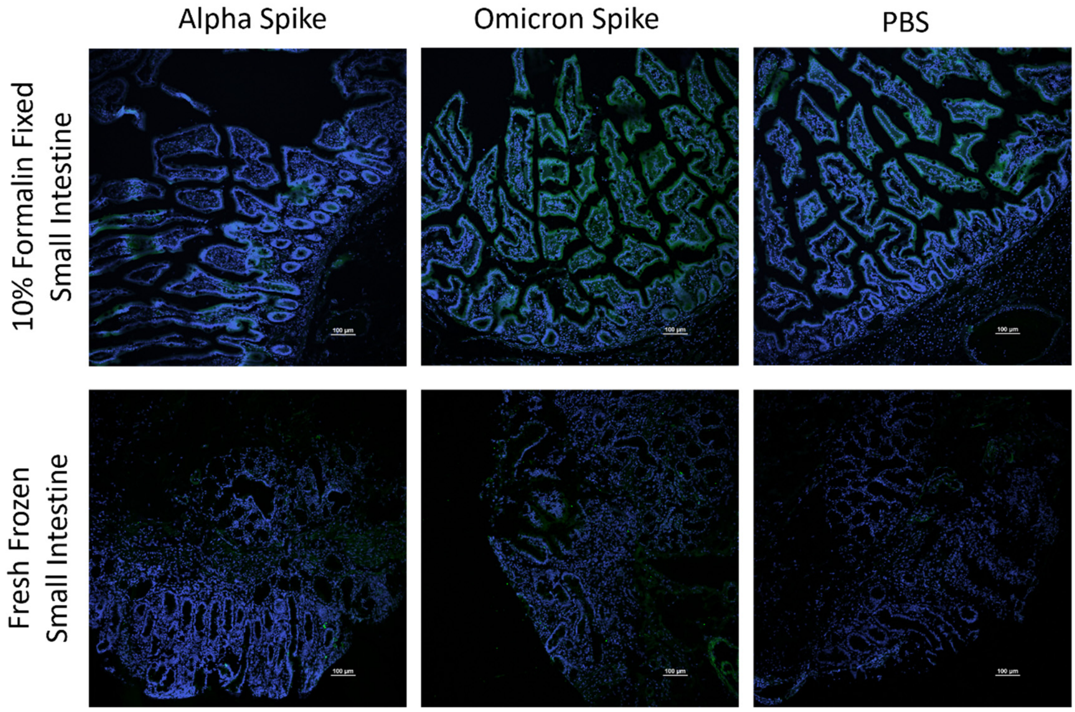

Supplementary Figure S3. Fluorescently labeled spike protein fails to bind to bind specifically to fresh-frozen small intestine or 10% formalin fixed small intestine (the positive control for these studies). Fixed and fresh-frozen sections were incubated with either GFP-labeled Alpha Spike (left), GFP-labeled Omicron Spike (middle) or PBS negative control (right) overnight with DAPI. Diffuse green nonspecific signal background was seen at high laser power without spike addition in PBS control formalin fixed section (upper right panel) and in Omicron spike (upper middle panel). No specific signal was seen in any fresh frozen tissues examined (lower panels). This indicates a lack of spike binding in either 10% formalin (top) or fresh frozen (bottom) tissue sections. This contrasts with robust staining for ACE2 in these sections shown in Figure 1 in the main article.

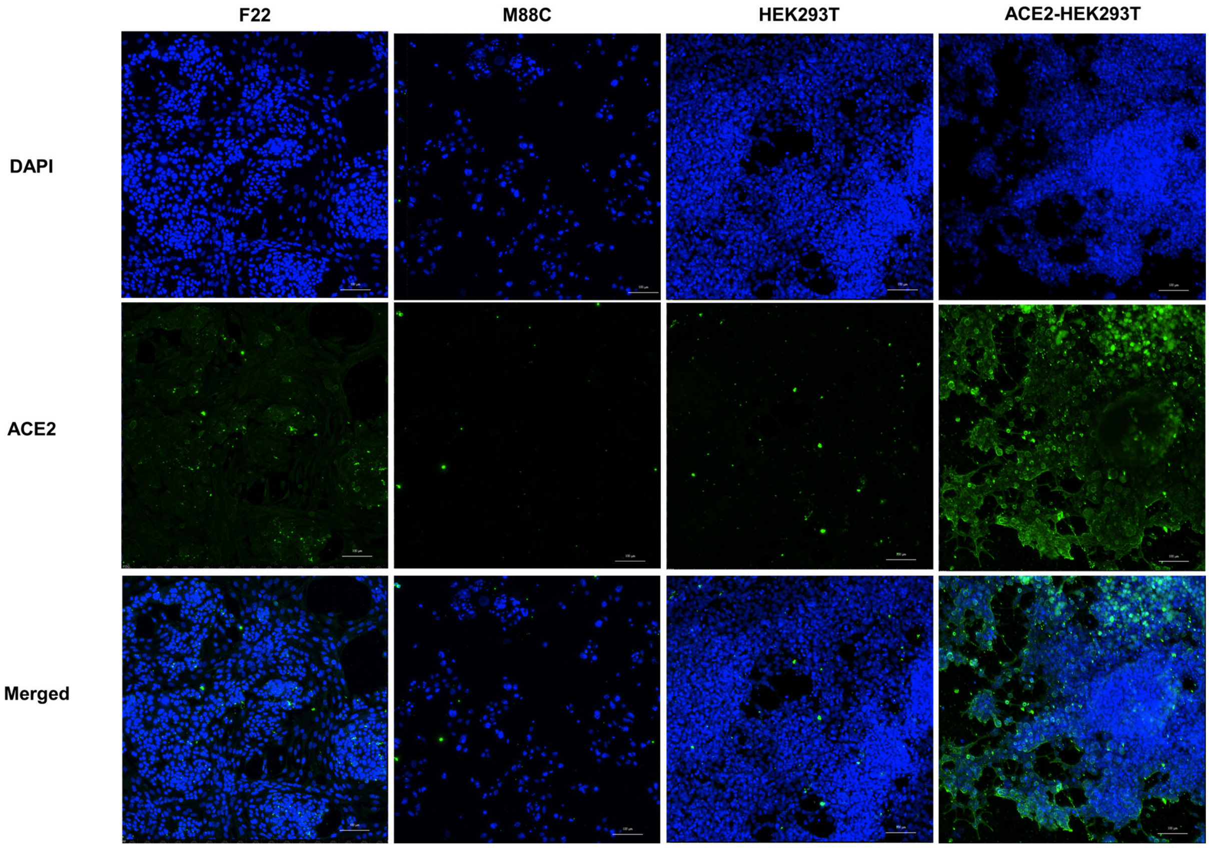

Supplementary Figure S4. ACE2 expression is minimal/absent in salivary epithelial cell preparations. Representative immunofluorescence images of salivary gland cell cultures from two patient specimens. Nuclei are labeled with DAPI (blue) and ACE2 immunoreactivity is shown in green. Across all conditions examined, salivary epithelial cells exhibit little to no detectable ACE2 signal, with only rare, sparse puncta observed at background levels. In contrast, bright green signal (right panels) confirm robust staining in ACE2-HEKT cells. Merged images (bottom row) further demonstrate the absence of significant ACE2 co-localization with salivary epithelial cells. Scale bars as indicated. These data indicate that salivary gland epithelial cells in this system do not express appreciable levels of ACE2 protein under the conditions tested.
